# Paradigm of Tunable Clustering Using Binarization of Consensus Partition Matrices (Bi-CoPaM) for Gene Discovery

**DOI:** 10.1371/journal.pone.0056432

**Published:** 2013-02-11

**Authors:** Basel Abu-Jamous, Rui Fa, David J. Roberts, Asoke K. Nandi

**Affiliations:** 1 Department of Electrical Engineering and Electronics, The University of Liverpool, Brownlow Hill, Liverpool, United Kingdom; 2 National Health Service Blood and Transplant, Oxford, United Kingdom; 3 The University of Oxford, John Radcliffe Hospital, Oxford, United Kingdom; 4 Department of Mathematical Information Technology, University of Jyväskylä, Jyväskylä, Finland; National Institute of Environmental and Health Sciences, United States of America

## Abstract

Clustering analysis has a growing role in the study of co-expressed genes for gene discovery. Conventional binary and fuzzy clustering do not embrace the biological reality that some genes may be irrelevant for a problem and not be assigned to a cluster, while other genes may participate in several biological functions and should simultaneously belong to multiple clusters. Also, these algorithms cannot generate tight clusters that focus on their cores or wide clusters that overlap and contain all possibly relevant genes. In this paper, a new clustering paradigm is proposed. In this paradigm, all three eventualities of a gene being exclusively assigned to a single cluster, being assigned to multiple clusters, and being not assigned to any cluster are possible. These possibilities are realised through the primary novelty of the introduction of tunable binarization techniques. Results from multiple clustering experiments are aggregated to generate one fuzzy consensus partition matrix (CoPaM), which is then binarized to obtain the final binary partitions. This is referred to as Binarization of Consensus Partition Matrices (Bi-CoPaM). The method has been tested with a set of synthetic datasets and a set of five real yeast cell-cycle datasets. The results demonstrate its validity in generating relevant tight, wide, and complementary clusters that can meet requirements of different gene discovery studies.

## Introduction

The main aim of conventional clustering is to group data points in a given dataset into clusters such that points belonging to one cluster are similar to each other while dissimilar to the points belonging to the other clusters according to some criterion [1].

Many methods have been introduced in the literature to tackle this problem such as self-organising maps (SOMs) [2], [3], [4], k-means [5], hierarchical clustering [6], self-organising oscillator networks (SOONs) [7], [8], fuzzy clustering [9], information-based clustering [10], and others. Each of these methods makes implicit assumptions about the nature of clusters and different clustering techniques give different results with the same dataset. Furthermore, the same method with different parameters or even the same parameters over different runs give different results and none of the methods gives the best results for all types of datasets.

One way to enhance the robustness of clustering is to combine results from many clustering experiments in clustering ensembles. Although classifier ensembles have been successful for supervised classifiers, combining results from different clustering experiments has been difficult as unsupervised clustering, where there are no identifying labels for the clusters, has no straightforward mapping between any specific cluster from one clustering experiment and its corresponding cluster from another experiment. Moreover, different clustering results might give different numbers of clusters while the correct number of clusters is unknown [11].

The main steps for most of ensemble clustering approaches are the “generation step” and the “consensus function step” [11]. In the generation step, different partitions (clustering results) are generated by using different clustering methods, initialisation parameters, subsets of the dataset or representations of data points in the dataset. Once the partitions are generated, they are fed to the consensus function which assigns data points in a consensus (final) partition.

Consensus functions can be generally classified into two main classes; data points co-occurrence and median partitions. Data points co-occurrence depends on the frequency of the appearance of a data point in a certain cluster or with another data point to build the final consensus partition. Some of the methods that belong to this class are relabeling and voting [12], [13], [14], co-association matrix [15], graph-based and hypergraph-based methods [16], [17], [18], and weighted kernel consensus functions [19], [20], [21]. Median partition methods formulate the problem as an optimisation problem. For *R* partitions {*P* ^1^ … *P^R^*}, the optimal consensus partition P^*^ is the one which is the most similar to all of them. This can be written mathematically as in equation (eq. 1):
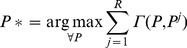
(eq.1)


where Γ(.,.) measures the similarity between any two partitions. This optimisation problem has been noted as an NP-complete problem [22], and some of the approaches in the literature that aim at solving it are non-negative matrix factorisation [23], [24], kernel-based methods [11], genetic algorithms [11], simulated annealing [22], and greedy algorithms [22].

In these methods, the final consensus partition assignment of data points is exclusive, i.e., no points are unassigned and no points are assigned to multiple clusters. This is a severe drawback, as in some cases, one gene product may participate in many processes and needs to be mapped to different functional clusters simultaneously [25]. It is also relevant that microarray datasets usually include the expressions of tens of thousands of genes while the relevant genes to the target problem are significantly smaller, usually of the order of hundreds or so [26].

Many gene discovery methods require zero false-positive assignments so that gene studies are focused. On the other hand, some studies might look for all of the genes that might belong to a certain cluster. This requires zero false-negative assignments even if few irrelevant genes are contained in the way.

One more issue, neglected in the literature, is to combine the clustering results of different clustering methods as well as to combine the results of clustering the expression profiles of the same set of genes from different microarray datasets. Obtaining a set of genes that are consistently co-expressed in different microarray datasets and when viewed by different clustering methods rather than being co-expressed in some of them and differently expressed in others is expected to benefit gene discovery research.

In this paper, we propose a novel ensemble clustering method, Binarization of Consensus Partition Matrices or Bi-CoPaM, which combines the results of clustering a set of genes by using different clustering methods and/or different microarray datasets into a single consensus result. We also demonstrate this tunable clustering method can solve the problem of different clustering or assignment requirements for different purposes. The main steps of the proposed algorithm include partitions generation, relabelling, fuzzy consensus partition matrix (CoPaM) generation, and its binarization. The new method exploits the information originated from different clustering results by generating a fuzzy CoPaM which characterises the membership (confidence level) of each data point in all the clusters. Crucially, the proposed binarization step can tune the generated clusters’ tightness to obtain tight clusters while leaving many genes un-assigned or to obtain wide clusters which overlap, or to obtain complementary clusters which assign each gene once and only once.

## Methods

This section describes the principles of the proposed method Bi-CoPaM [27], [28]. The problem is to group *M* data objects into *K* clusters. In the context of gene clustering, genes represent objects which are clustered based on their expression profiles. Clustering is carried out over *R* different experiments which generate *R* different partitions {*P* ^1^, … , *P^R^*}. The goal is to find the final consensus partition P^*^ which relaxes the conventional partitioning constraints by allowing some points to be assigned to multiple clusters at the same time or to be not assigned at all in a way which best reflects the information provided by the partitions.

The four main steps of the algorithm are:

- Partition generation: *R* different clustering experiments are carried out to generate *R* partitions. Each resulting partition *P^j^*, for *j*  =  1 … *R*, is presented in the form of a partition matrix 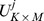. The properties of this matrix and the details of partitions generation are detailed later on in a separate subsection.
- Relabelling: The clusters in the generated partitions are relabelled such that corresponding clusters from different partitions are aligned.
- Fuzzy consensus partition matrix generation: Relabelled partition matrices are averaged to generate the CoPaM.
- Binarization: This is the primary novelty of the proposed method. The final partition (which allows for every data point, to belong to a single cluster or to multiple clusters or to no cluster at all) is obtained from the CoPaM.

### Partition Generation

To group *M* data points into *K* clusters, *R* clustering experiments are carried out to generate *R* partitions. These experiments can use different clustering methods on the same data, or same clustering method with different parameters on the same data, or different clustering methods on different data, and many combinations thereof. Each clustering partition can be presented in the form of a fuzzy partition matrix. The matrix 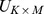 is a 2D matrix with *K* rows representing the clusters and *M* columns representing the data points. Each element of the matrix 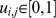 represents the membership value of the *j^th^* point in the *i^th^* cluster. A value of zero means that this point does not belong to this cluster at all, and a value of one means that it fully belongs to it.

In crisp clustering, where each point belongs exclusively to one cluster, the values of the elements are strictly either zero or one. In the general case of fuzzy clustering, the elements can have any value between zero and one inclusively. The following conditions must be satisfied by the fuzzy partition matrix [29]:

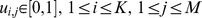

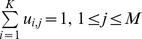

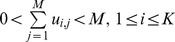

In [12], [16], they use the transpose of this definition.

### Relabelling

The partitions of different clustering experiments over the same dataset are not guaranteed to be aligned, i.e. for a *K*-cluster problem, the *i^th^* cluster of one of the partitions might correspond to any of the {1 … *K*} clusters in another partition. This is a labelling correspondence problem [11] which is an NP-complete combinatorial problem [14]. Relabelling reorders the clusters in each of the partitions such that they are all aligned.

Relabelling a partition matrix *U* to be aligned with a reference partition matrix 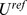 aims at finding a matrix 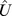 which represents one of the permutations of the rows of *U* such that its similarity to 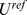 is maximised, which can be expressed as 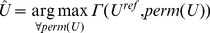
(eq.2)


Where *perm(U)* is a permutation of the rows of *U*, and Γ(.,.) is the similarity measure.

**Figure 1:**
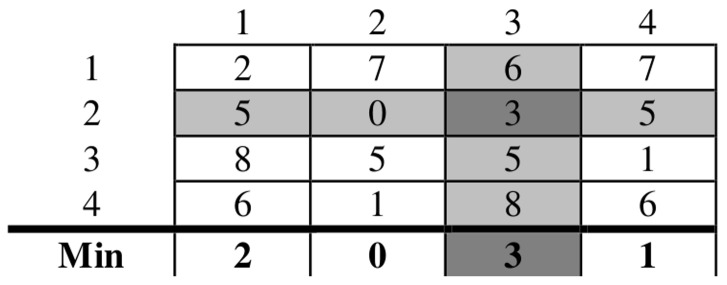
Sample pairwise similarity matrix for fuzzy partition matrices’ rows relabeling. Each element in this sample pairwise matrix measures the similarity between a cluster from one clustering result and a cluster from another clustering result. In the min-max relabelling approach, the minimum value of each column is calculated, as shown in the row below the matrix, and then the maximum of these minima is considered. The maximum of the minima is shaded in dark gray and the clusters corresponding to the row and the column containing this value are matched. This row and this column are then removed and the process is repeated until each cluster in the first result is matched with a cluster from the second result.

The size of the search space is *K*!, which makes brute force search impractical for not so large values of *K*, the case in which different heuristic approaches can be used such as the maximum greedy (MG), the mean enhanced greedy (MEG), and the single mean enhanced greedy (SMEG) algorithms [30]. In this paper we follow the same logic of the MEG and the SMEG algorithms. We follow a *min-max* approach as detailed below:

- A dissimilarity matrix 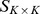 is constructed with pairwise Euclidian distances between the rows (clusters) of the matrix *U* and the rows of the reference matrix 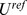.
- The minimum value in each of the columns is found.
- The maximum value of these minima is identified, then the rows (clusters) from *U* and 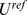 which correspond to this dissimilarity value are mapped.
- A sample of relabelling is shown in **Figure 1**. The result of this step is the assignment of the clusters that correspond to the 2^nd^ row and the 3^rd^ column.
- The row and the column which show the aforementioned value are deleted from the similarity matrix.
- If all of the *K* rows from *U* and 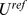 are mapped, the algorithm terminates, otherwise it goes back to step (b) with the reduced similarity matrix.

### CoPaM Generation

Formally, there are *R* partitions generated by *R* different clustering experiments. All of these experiments group the same set of *M* data points into *K* clusters. Let the *r^th^* partition be generally represented by the fuzzy partition matrix 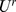 which has *K*×*M* elements 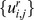. The aim is to reorder the rows (clusters) of all of the partition matrices to be aligned, then to find the element by element mean of all of them to generate the CoPaM 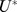.

One method considers the first partition as the reference and relabels the others to generate the CoPaM. Another method suggests that an intermediate fuzzy CoPaM 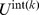 is initialised with the values of the first partition *U* ^1^, and then the other partitions are relabelled and fused with this intermediate matrix one by one while considering it as the reference at each step [12]. The later suggestion is considered in this paper.

Let 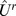 be the relabelled partition matrix of the partition 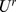, and 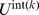 be the intermediate CoPaM after the *k^th^* stage, i.e. after relabelling and fusing the partitions 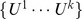. Let the function 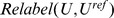 denote relabelling the partition matrix *U* by considering 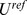 as the reference partition. Equation (eq. 3) shows how the intermediate partition matrix can be calculated by the normal approach and the recursive approach:
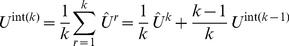
(eq.3)


Generating the fuzzy consensus partition matrix (CoPaM) is achieved by following the algorithm shown in the following steps:

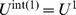

- for *k*  =  2 to *R*

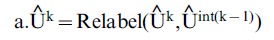


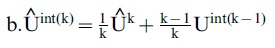

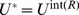

### Binarization

The conventional exclusive assignment in clustering is relaxed in order to generate a consensus binary partition 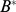 from the final CoPaM 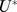. The relaxed consensus binary partition 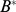 is a pseudo-partition matrix with *K* rows for the clusters and *M* columns for the data points. Each element 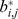 represents the membership of the *j^th^* point in the *i^th^* cluster and satisfies the following conditions:

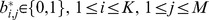

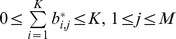

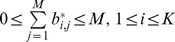

The first condition guarantees that the matrix is binary. The second and the third conditions formulate the relaxed nature of this matrix. From the second condition, a certain data point may not be assigned at all (the summation is zero) or assigned to one and only one cluster (the summation is one) or assigned to more than one cluster at the same time up to *K* (the summation is greater than one). From the third condition, any cluster is allowed to be empty or to include all the data points (in this case, other clusters are not necessarily empty because multiple assignments are allowed).

Different binarization techniques are proposed to allow for different eventualities. Two measurements are monitored for each of the techniques - 1) 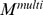 (the number of points assigned to more than one cluster), and 2) 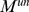 (the number of points not assigned to any of the clusters).

Six binarization techniques, namely Intersection Binarization, Union Binarization, Maximum Value Binarization, Value Thresholding Binarization (α-cut), Top Binarization, and Difference Thresholding Binarization, are given below.

#### Intersection Binarization (IB)

This is the strictest binarization technique where a data point is assigned to a cluster if *all* of the partitions map this data point to that cluster. This is formulated as
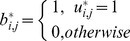
(eq.4)


This technique results in 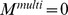 and 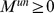.

#### Union Binarization (UB)

This is the loosest binarization technique where a data point is assigned to all the clusters to which at least one partition assigned it. It is defined as
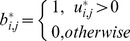
(eq.5)


This technique results in 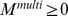 and 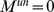.

#### Maximum Value Binarization (MVB)

Each data point is assigned to the cluster to which its maximum membership value points. If more than one cluster share the same maximum value, it is assigned to all of them. It is formulated as
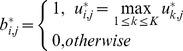
(eq.6)


This technique results in 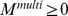 and 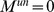. The value of 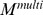 is usually very small and is likely to reach 0.

#### Value Threshold Binarization (α-cut) (VTB)

Each data point is assigned to all of the clusters in which its membership values are not less than a threshold (α), i.e.
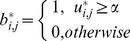
(eq.7)


This technique results in 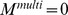 for 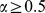 and 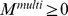 for 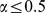. In general, 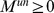 and it increases as α increases.

#### Top Binarization (TB)

This is a relaxed version of the MVB technique such that each data point is assigned to the maximum membership value cluster and to all of the clusters in which its membership values are within a certain difference (δ) bellow the maximum, i.e.
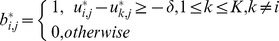
(eq.8)


This technique results in 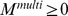 and 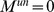. The value of 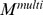 is larger than that of the MVB technique and increases as the value of δ increases.

#### Difference Threshold Binarization (DTB)

This is a stricter version of the MVB technique in that each data point is assigned to the maximum membership value cluster only if the value of the closest competitor cluster is at least as far from the maximum as a predefined difference (δ) i.e.
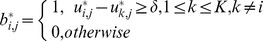
(eq.9)


For 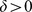, this technique results in 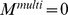 and 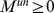. The value 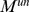 increases as the value of δ increases.

### Binarization-Related Issues

#### Binarization Tracks

Careful scrutiny of the way in which the six binarization techniques control the tightness and wideness of the clusters leads to classifying them into two classes of techniques. We refer to these two classes as binarization *tracks*. Each of the two tracks starts with very wide clusters and then tightens them gradually to reach very tight clusters.

The first track consists of the TB, MVB and DTB techniques. The MVB technique generates complementary clusters where each data point is assigned exclusively to one and only one cluster. The very rare exception is when more than one cluster share exactly the same maximum fuzzy membership value. In this case, the point is assigned to these clusters simultaneously. MVB is equivalent to both TB with δ = 0 and DTB with δ = 0. Increasing the parameter of the TB or the DTB technique moves the clusters away from this central MVB case towards wider or tighter cases, respectively. The widest case is at TB with δ = 1, and the tightest case is at DTB with δ = 0. We refer to this track as the TB-MVB-DTB track of binarization.

The second track consists of the other three techniques, UB, VTB and IB. It generates its widest clusters by the UB technique; this is equivalent to using VTB with α = ε, where ε is a very small positive real number just bigger than zero. Increasing the value of this parameter tightens the clusters gradually until they reach their tightest possible case at α = 1, which is equivalent to the IB technique. Note that this track has no case equivalent to the MVB technique of the first track which generates complementary clusters. We refer to this track as the UB-VTB-IB track of binarization.

The main philosophical difference between the two tracks is in the bases upon which they judge the assignments of data points to the clusters. The UB-VTB-IB track merely considers the absolute fuzzy membership value of the data point in the corresponding cluster. For example, if a data point belongs to a certain cluster with a membership of 0.4 and the VTB parameter α was chosen to be 0.4 (or less), then this data point is assigned to this cluster regardless of its membership in the other clusters.

On the other hand, the TB-MVB-DTB track considers the competitiveness of the clusters on that single data point. The TB technique assigns that data point to that cluster as long as it is not lower than the maximum membership by more than the parameter δ, MVB assigns it to that cluster if it has the maximum membership in it, and DTB assigns it to that cluster as long as there is no competing cluster with a membership value closer than the parameter δ. That is how the TB, MVB and DTB techniques respectively generate wide, complementary and tight clusters while considering the competitiveness of the clusters over the same data point.

#### Tunable Binarization

The different nature of the parameters of the VTB, TB and DTB techniques imposes different ways of using them. Although in principle the three parameters can be spanned from zero to one, some cases are unreasonable and should not be considered in practice. For example, the case of VTB with α = 0 results in the trivial case where each single data point is assigned to all of the *K* clusters. Thus, practical α values would be from any positive real number larger than zero to one. The TB parameter δ needs careful attention, as high δ values result in the assignment of many data points to some clusters although their membership values in them are zero. The extremest case is at TB with δ = 1 where each data point is assigned to all of the clusters. So, the TB’s δ value should be spanned from zero to a modest value between zero and one depending on how fast clusters grow corresponding to δ’s increase. The DTB’s parameter δ can be spanned from zero to one without, in general, resulting in unreasonable results.

That being said, different binarization combinations (techniques and parameters) might suit different applications and/or might reveal different information from the data. For example, using the IB, the DTB or the VTB with high values of α can find the tightest clusters, identifying the data points that are relatively clearly assigned to a cluster. Using the UB, the VTB with small values of α or the TB with high values of δ might result in wide clusters, containing points that are likely to belong. This can be useful in identifying the data points that have multiple roles, and different applications might find different meanings for this, e.g. in gene clustering, these genes (data points) perhaps participate in different biological functional groups (clusters).

MVB and TB with small values of δ are well adapted in finding complementary clusters with few multi assignments, which is closer to crisp clustering. The value of δ can be tuned for the most suitable results for a particular problem.

## Synthetic Datasets Analysis and Results

This section describes the synthesis of a set of 60 synthetic cyclic gene-expression microarray datasets, the Bi-CoPaM experiments carried out over them, and their results.

### Datasets Synthesis

Sixty synthetic microarray datasets with varying levels of noise were generated to test and validate the Bi-CoPaM method. Each dataset consists of the expression of 450 genes over 24 time points and were synthesised to show cyclic sinusoidal patterns that cover two complete cycles over the given time points. The 450 genes, as synthesised, belong to five different groups that are characterised by their patterns’ phase shift values.

The method of the synthesis was proposed in [31], [32] and the specific synthesis equation used in this research is:
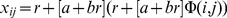
(eq.10)

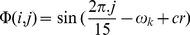
(eq.11)


Where 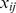 is the expression value of the *i^th^* gene at the *j^th^* time point, each instant of *r* in (eq. 10) and (eq. 11) is an independent random number from the standard normal distribution *N*(0,1), *a* controls the magnitude of the sinusoid and it is fixed to 3.0 here, *b* controls the random component added to the magnitude, *c* controls the random component added to the phase, and 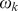 is the phase shift of the *k^th^* cluster, i.e. the cluster to which the *i^th^* gene belongs.

The parameters *b* and *c* were varied to generate 60 datasets with varying levels of noise. The used values for these parameters were:
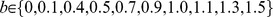


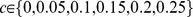
.

The goal of varying *b* and *c* is merely to vary the noise level, and it is more intuitive to view the resulting datasets by using a common metric such as the signal-to-noise ratio (SNR). Thus the 60 datasets were mapped from the (*b – c*) 2D space to the corresponding SNR.

SNR measures the ratio of the power in the pure signal to that in the noise. The pure signal in this case is generated by using the equation (eq. 10) with the random components set equal to zero. The noise is calculated by subtracting the pure signal from the noisy one. If 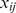 and 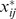 are the actual and pure expression values of the *i^th^* gene (out of *M* genes) at the *j^th^* time point (out of *N* time points) respectively, then the average SNR value for this dataset is calculated by the equation:
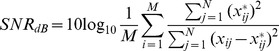
(eq.12)


The lowest and the highest SNR values are 2.96 dB and 7.31 dB, which are pretty challenging. SNR values have stronger dependence on *b* (affecting amplitude) and weaker dependence on *c* (affecting phase). Hereinafter, the SNR values will be used to identify the corresponding dataset, without reference to *b* and *c*.

### Experimental Clustering Procedure

Four clustering methods with different configurations were applied over the datasets to generate sets of clustering partitions. The resulting CoPaM matrix was binarized using different techniques with different parameters to generate the final partitions. Table 1 lists the details of the clustering experiments. These same experiments were applied over the real yeast datasets whose details are provided in another section later on in this paper. The tenth experiment, SOON clustering, used customised parameters for different datasets.

**Table 1:** Clustering experiments.

No.	Method	Parameters
1	k-means	Empty clusters were dropped and Kaufman deterministic initialisation [5] was used.
2	SOMs	Batch mode learning, 2D hexagonal grids and bubble neighbourhood (Repeated 20 times then combined)
3	SOMs	Batch mode learning, 2D hexagonal grids and Gaussian neighbourhood. (Repeated 20 times then combined)
4	HC	Single linkage
5	HC	Complete linkage
6	HC	Average linkage
7	HC	Centroid linkage
8	HC	Ward linkage
9	HC	Median linkage
10	SOON	For synthetic dataset*,#; *b*∈{0.1, 0.5, 1, 10, 20, 40, 60, 80, 100, 120}; *C_E_*∈{0.1, 0.12, 0.14, 0.16, 0.18, 0.2, 0.3, 0.4}; *d_0_*∈[3.6∶0.2∶6.0]
11	SOON	For yeast datasets*,#; *b*∈{0.1,1,50,100}; *C_E_*∈{0.1∶0.02∶0.2}; *d_0_* (cdc28)∈[4.5∶0.05∶5.0]; *d_0_* (cdc15)∈[6.25∶0.05∶6.75]; *d_0_* (alpha)∈[5.3∶0.02∶5.5]; *d_0_* (alpha30)∈[6.3∶0.05∶6.8]; *d_0_* (alpha38)∈[6.1∶0.05∶6.6]

* *b* is the constant which controls the concavity of the mapping function, *C_E_* is the constant of excitation, and *d_0_* is the radius of the clusters [8].

# For each of the two cases; the synthetic and the yeast ones, The results of using all of the possible parameters’ combinations were combined into one partition.

These clustering methods were chosen as they explore the data points differently; SOMs exploit the topological distribution while SOON focuses on intensity levels; HC is greedier, SOON does not require prior knowledge of the number of clusters, and k-means gives reasonable spherical clusters, and so on. This is expected to increase diversity which is important for binarization.

The partitions’ generated by these different clustering methods for each of the 60 synthetic datasets were combined in a single CoPaM. Then, each of the 60 produced CoPaMs was binarized using the six binarization techniques IB, UB, MVB, DTB, VTB and TB with different parameters’ values.

### Results

With respect to these datasets, four different objectives are addressed by Bi-CoPaM – 1) obtain clusters with maximum correct assignments, 2) obtain tight clusters with minimum false-positives, 3) obtain wide clusters with minimum false-negatives, and 4) roughly detect the level of noise.

False-positives are the genes that are assigned incorrectly to clusters. If a gene is assigned to multiple clusters where all of them are wrong, this gene adds the value of one to false-positives’ count. If a gene is assigned to multiple clusters and some of them are correct, this gene adds the value of *(Number of wrong assignments) / (Number of all assignments)* to the false-positives’ count. This guarantees the same weight of contribution from each gene in calculations.

#### Maximisation of Correct Assignments

**Figure 2:**
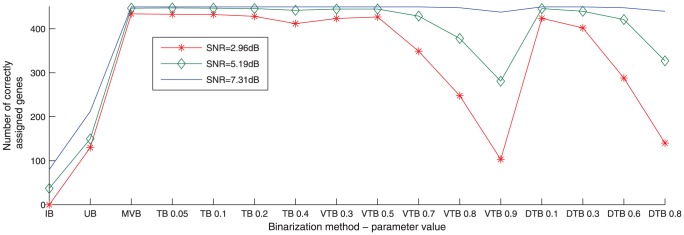
Correctly assigned genes. The number of correctly assigned genes at the y-axis is plotted versus the 16 binarization configurations at the x-axis for three representative synthetic datasets out of 60. It should be noted that the binarization configurations are not entirely ordered according to their tightness.

We assessed the performance of different binarization techniques in forming clusters which maximised the number of correct assignments, while minimising the number of false-assignments. Three representative sets out of the 60 available ones were picked; the best (SNR = 7.31 dB), the worst (SNR = 2.96 dB), and the median (the 31^st^ best one, SNR = 5.19 dB). The number of correctly assigned genes versus each of the 16 binarization configurations for these datasets is plotted in **Figure 2**.

The number of correctly assigned genes in datasets with higher SNR values is clearly higher, as expected. Indeed, at the maximum SNR, all 450 genes were correctly assigned at ten of the 16 binarization configurations.

The second observation is that the best binarization configurations for this purpose are those that are closer to crisp clustering, i.e. the ones that result in the minimum amount of multi-assigned and un-assigned genes. This is expected theoretically because un-assignments in this case will always result in false-negatives, and multi-assignments will always result in false-positives. The closest binarization technique to crisp clustering is the MVB as it results in no un-assignments and minimum multi-assignments, which reaches absolute zero in most of the times. TB with δ = 0.05, VTB with α = 0.5, and DTB with δ = 0.1 are the next closest binarization configurations to crisp clustering as they allow for no more than a few un-assigned or multi-assigned genes.

#### Minimisation of False-Positives

The minimisation of the number of false-positive genes is obtained by using the binarization configurations which tighten the clusters and throw the uncertain genes out of all clusters. The MVB lies between the techniques that result in multi-assigned genes and un-assigned genes. VTB and DTB tighten the clusters more as their parameters α and δ are increased respectively. IB is the strictest binarization technique and it is equivalent to VTB with α = 1.0, and to DTB with δ = 1.0, so this technique results in the absolute possible minimum false-positives for any dataset.

We propose an index to calculate the ratio of the false-positives to the number of assigned genes. Equation (eq. 13) formulates the false-positives index (*FPI*), which needs to be minimised.
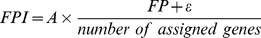
(eq.13)


The addition of a small positive number ε to the number of false positives (*FP*) ensures that 0/450 is better than 0/400, because obtaining false-positive-free results while assigning more genes to the clusters is better. The factor *A* provides a convenient scaling.

**Figure 3:**
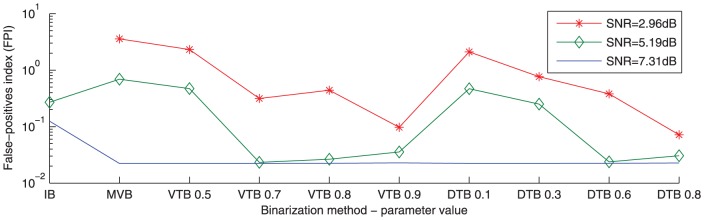
False-positives index (*FPI*). False-positives index (*FPI*) is plotted in log scale versus a subset of binarization configurations for three representative synthetic datasets out of 60. It should be noted that the binarization configurations are not entirely ordered according to their tightness.

**Figure 3** plots the *FPI* for three representative datasets, same as in **Figure 2**, at different binarization configurations with ε = 0.1 and *A* = 100. Configurations which result in many multi-assigned genes show large numbers of false-positives, thus they are not included in this plot. Note that the very strict IB can result in totally empty clusters with 450 unassigned genes, resulting in division by zero in *FPI* values, and are shown in the Figure as missing values.

As shown, tightening the clusters by increasing the binarization parameters’ values decreases *FPI*, but after a certain point, although lower numbers of false-positives are obtained, a lot of genes are lost from the clusters with a resulting increase of *FPI* values.

For example, the dataset with (*SNR* = 5.19 *dB*), reaches its minimum *FPI* at the threshold α = 0.7 of the VTB technique. If the threshold is increased to 0.8 or 0.9, more genes are lost from the clusters with no false-positives reduction, hence, higher *FPI*. In general, noisier datasets tend to need tighter configurations in order to reach their minimum values of *FPI*.

Calculating *FPI* is feasible here because of the existence of the ground truth, and it is used here to validate the usage of the Bi-CoPaM method. In real applications, the biologist might prefer to get very tight clusters even if most of the genes are unassigned at all or less tight clusters. This choice depends on the application, and this experiment provides the proof of principle for this flexibility.

#### Minimisation of False-Negatives

The third objective of using Bi-CoPaM is to obtain wide clusters which minimise the number of false-negative genes. To get an optimum result, the minimum number of false-negatives should be obtained while minimising the number of multi-assigned genes. For this purpose, a false-negatives index (*FNI*), which should be minimised, is introduced here (eq. 14):


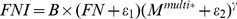
(eq.14)

Where *FN* is the number of false-negative genes. 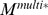 is the number multi-assignments such that if a gene is assigned to two clusters simultaneously it is counted once, if it is assigned to three clusters it is counted twice, and if it is only assigned to one cluster or to no clusters, it is not counted at all. *B*, 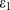, and 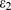 are used for the same reasons for which *A* and ε are used in the *FPI* index above. The parameter γ, which controls the relative influence of the number of multi-assigned genes to the *FNI* value compared with the influence of the number of false-negatives, can be chosen according to the researcher’s needs.

**Figure 4:**
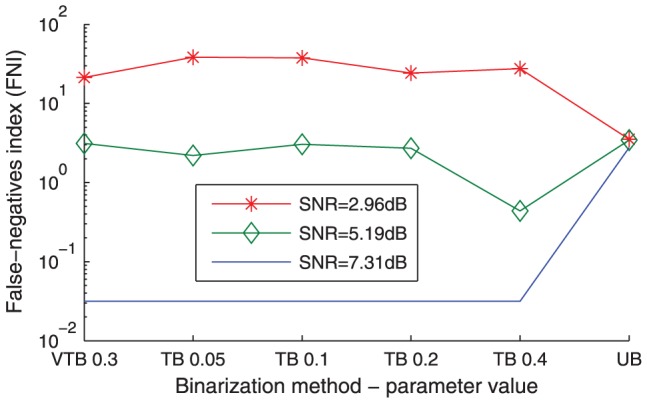
False-negatives index (*FNI*). False-negatives index (*FNI*) is plotted in log scale versus a subset of binarization configurations for three representative synthetic datasets out of 60. It should be noted that the binarization configurations are not entirely ordered according to their tightness.

**Figure 4** plots the *FNI* profiles of three representative synthetic datasets over six binarization configurations which result in multi-assigned genes, with *B* = 1, 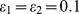, and *γ* = 0.5. Recall that as the value of δ for TB technique increases, looser clusters are generated. The loosest technique is UB, which is equivalent to TB with δ just less than one, and it gives the maximum 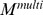.

Noisier datasets tend to provide wider clusters when minimising the *FNI* value, as wider clusters are likely to include all genes that belong to them giving rise to low false-negatives. The *FPI* and *FNI* analyses using synthetic datasets aim at validating the Bi-CoPaM method and are not provided as validation tools for real datasets.

#### Noise Level Effect and Estimation

The fourth possible objective of the Bi-CoPaM method is rough noise level detection. This can be achieved by monitoring the rate of increase in the number of un-assigned and / or multi-assigned genes while tightening and / or widening the clusters, respectively.

**Figure 5:**
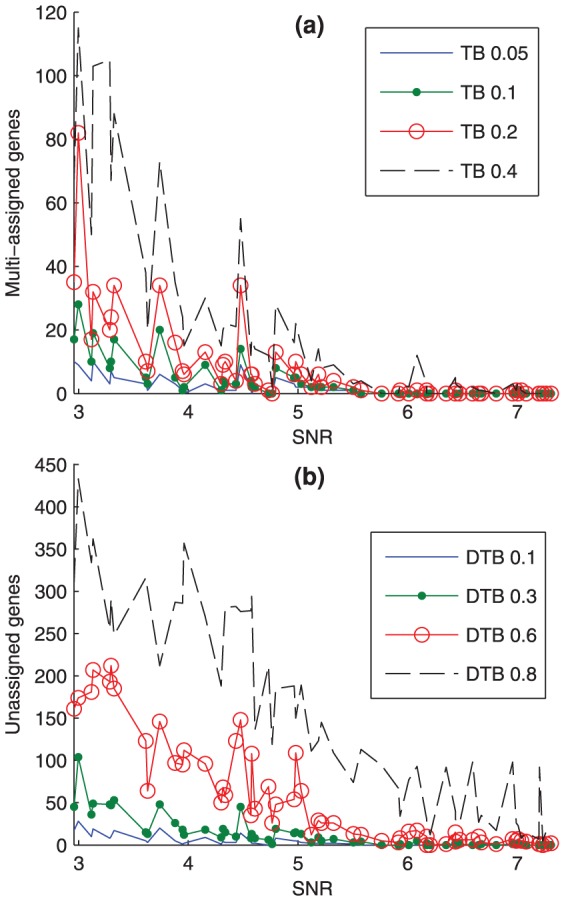
SNR effect over the number of multiply assigned and unassigned genes. (a) The number of multi-assigned genes is plotted over the 60 SNR values in four cases of wide clusters generated by using the TB technique. (b) The number of unassigned genes is plotted over the 60 SNR values in four cases of tight clusters generated by using the DTB technique. Note that there are no multi-assigned genes in tight clusters as there are no unassigned genes in wide clusters.

**Figure 5(a)** shows the numbers of multi-assigned genes (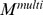) while adopting TB with δ = 0.05, 0.1, 0.2, and 0.4 over the 60 synthetic datasets ordered by their SNR values. It can be noticed for a particular dataset, i.e. a particular SNR value in this plot, the rate of increase in 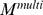 while increasing δ is usually higher for noisier ones. Also, while comparing different datasets with each other, the values of 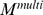 tend to decrease for purer datasets, i.e. for higher SNR datasets.

**Figure 5(b)** shows the numbers of unassigned genes (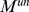) while adopting DTB with δ = 0.1, 0.3, 0.6, and 0.8 over the 60 synthetic datasets. The same observation seen in the behaviour of 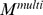 while widening the clusters can be seen for 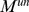 while tightening the clusters. Thus, either approach can be used to estimate the approximate, though not the definitive, level of noise in a datasets, or to compare noise levels in two datasets.

This analysis is correct when **Figure 5** is examined globally. If we zoom into a local neighbourhood of close SNR values, we would observe some fluctuation in the levels of multi-assigned and unassigned genes. Careful investigation of this observation shows that, in many cases, it is caused by the differences in the effects of varying the noise imposed on the amplitude and the noise imposed on the phase. Recall from the model in equation (eq. 10) that the two parameters *b* and *c* were used to vary the noise in the amplitude and the phase, respectively. The SNR values summarise the overall noise level globally, but the effects of varying the phase noise seem to be more severe than when varying the amplitude noise, even when the resulting SNR values are close.

For example, the three consecutive datasets represented by the SNR values 4.44, 4.48, and 4.57 dB, when clustered and binarized by TB with δ = 0.4, showed 21, 55 and 12 multiply-assigned genes, respectively. This is seen as an obvious peak in **Figure 5(a)** around those SNR values. The (*b*, *c*) pairs which were used to synthesise these three datasets are (1.1, 0), (1, 0.2) and (1.1, 0.05). Although their SNR values are very close, the (*c*) value which was used to generate the second dataset of them is significantly higher than in the other two, i.e. 0.2 versus 0 and 0.05. This indicates that some small variations in the phase can affect the purity of the clusters more than what is reflected by the corresponding variations in SNR values. Thus, the relation between the results and the noise level should be considered globally not locally.

Estimation an approximate noise level of a dataset might help to optimise the tightening and / or widening levels for a certain application. This claim can be justified by the *FPI* and *FNI* analysis above, and opens the possibility to design validation indices which apply this approximation of noise level to tune the binarization techniques based on the needs of the application.

### Random Periods Model (RPM) Synthetic Data Analysis

Liu and colleagues criticised the cell-cycle regulated genes’ models that do not consider the attenuation of cyclic expressions with time [33], such as the model which we have based our analysis on. They showed that the asynchrony which occurs between the cells in the culture results in an attenuation in the resultant expression profile. They then proposed a random-periods model (RPM) for cell-cycle regulated genes [33].

Thus, we provide an additional section of analysis in which we generate a more realistic synthetic dataset by the RPM model over which we apply the Bi-CoPaM method. The details of the RPM model, the generation of the synthetic dataset, the experimental procedure, and the results are included in the section “Random Periods Model (RPM) Synthetic Data Analysis” of File S1.

## Real Yeast Datasets Analysis and Results

This section discusses the analysis of five real yeast cell-cycle datasets. First, the five datasets are introduced. Then the experimental procedure is detailed, and finally the results are shown.

### Real Yeast Cell-Cycle Datasets

In [31], 384 yeast genes were identified as cell-cycle regulated genes whose expression profiles show periodic patterns over the cell cycle, which includes five main stages - early gap 1 (early G1) corresponding to the beginning of interphase, late gap 1 (late G1), synthesis (S), prometaphase (G2), and metaphase (M) [31], [34], [35], [36]. These 384 genes are expected to form five clusters depending on the stage in which their periodic patterns show peak values [31], [37].

Five budding yeast cell-cycle microarray datasets are considered in this study. Each dataset has gene expression values of more than 6000 genes that almost cover the entire yeast genome over two complete cell cycles. Differences among these five experiments are in the microarray technology and the biological preprocessing carried out over the yeast cells as well as the cell synchronisation method. Also, they differ in the number of time points at which the samples were taken as well as the time between them.

**Table 2** lists five datasets and some of their parameters. The first column shows the synchronisation method by which we uniquely refer to each of these datasets, the second column shows the year in which each of these datasets was made public, the third and the forth columns show the number of time points (samples) and the time between two consecutive time points respectively, the fifth column shows the maximum number of allowed missing values in a gene’s expression, and the sixth column shows the references.

**Table 2:** Budding yeast cell-cycle microarray datasets.

Synch. method	Year	Time points	Spacing (min)	Allowed missing values	Ref.
Cdc28	1998	17	10	3/17	[34]
Cdc15	1998	24	10*	6/24	[35]
Alpha	1998	18	7	5/18	[35]
Alpha-30	2006	25	5	1/25	[36]
Alpha-38	2006	25	5	1/25	[36]

* Five of the time spaces between these samples are 20 minutes instead of 10.

While dealing with multiple datasets, it is important to consider some tolerance in dealing with missing values so that fewer genes are filtered out of the analysis. In this study, we tolerate different numbers of missing values from different datasets depending on the total number of time points and on the quality of the dataset (newer are usually better). The upper limits for the number of allowed missing values in a gene’s expression to be considered in the analysis are included in the fifth column of **Table 2**.

Some 340 genes out of the original 384 genes were considered for clustering as their expression profiles do not exceed the upper limit of allowed missing values in any of the five datasets. The remaining missing values were replaced by ‘spline’ interpolation [38]; then the expression profiles were normalised by subtracting the mean and dividing by the standard deviation.

### Experimental Clustering Procedure

The 340 genes were clustered from each of the five datasets into five clusters by using the same setup that was used for the synthetic datasets. As can be seen in **Table 1**, the only difference is in the parameters of the SOON clustering method; this is due to the differences in the distribution between these different datasets.

For these real datasets, all of the yeast genes’ partitions generated by applying those different clustering methods to the five yeast datasets were combined into a single CoPaM (not five CoPaMs) which was binarized by using the six binarization techniques IB, UB, MVB, DTB, VTB and TB with different parameters’ values.

### Results

**Table 3** summarises the results of the comprehensive Bi-CoPaM clustering analysis over the 340 considered genes from the five datasets listed in **Table 2**. The genes were clustered in five clusters by the methods listed in Table 1, then combined and binarized by different configurations of binarization (techniques and parameters). The results in this table are grouped into two groups which show the gradual tightening of clusters from the very wide case towards the very tight case based on the two different tracks of binarization – the TB-MVB-DTB track and the UB-VTB-IB track.

**Table 3:** Assignment of genes from five real yeast datasets by Bi-CoPaM.

			Genes in clusters							Genes in clusters				
	Tech.	Param.	C1	C2	C3	C4	C5	Tech.	Param.	C1	C2	C3	C4	C5
Wide	TB	0.8	260	316	264	248	258	UB	-	328	340	320	296	329
	TB	0.4	129	198	122	100	109	VTB	0.1	139	194	127	92	205
	TB	0.2	92	149	82	52	65	VTB	0.2	97	161	96	62	98
	TB	0.1	82	139	72	47	48	VTB	0.4	68	130	57	39	26
Complementary	MVB	-	73	135	57	41	34	VTB	0.5	60	123	48	35	5
	DTB	0.1	62	128	55	36	15	VTB	0.6	43	109	39	25	1
	DTB	0.2	60	120	49	33	5	VTB	0.8	14	65	17	8	0
	DTB	0.4	38	102	38	24	1	VTB	0.9	8	49	7	0	0
Tight	DTB	0.8	11	49	10	0	0	IB	-	0	0	0	0	0
	**Track 1 (TB-MVB-DTB)**							**Track 2 (UB-VTB-IB)**						

#### The Five Resulting Clusters

The five resulting clusters are labelled by C1, C2, C3, C4, and C5 in Table 3. By analysing the patterns of the genes assigned to each of these five clusters and matching them with the five yeast cell-cycle stages, it is observed that C1 corresponds to the early-G1 stage, C2 to late-G1, C3 to S/G2, C4 to M, and C5 does not show consistent patterns and is not mapped to any of the stages.

#### Tightening / Widening Effects and Analysis

From the variations in the numbers of genes included in the five clusters at different levels of widening and tightening, the cluster C5 is found to be the noisiest as it loses its genes very quickly; only one gene exceeds the membership value of 0.6 in it (Table 3). This observation is consistent with remarks in the previous subsection.

On the other hand, the clusters C1, C2, and C3 preserve a fair number of their genes up to some of the tightest cases, i.e. DTB with δ = 0.8 and VTB with α = 0.9. This indicates that these are purer clusters whose genes have more consistency in different datasets as well as when clustered by different clustering methods. No genes survived in the extreme case of IB in any of the five clusters. This is because using more clustering methods with many different sets of parameters over many datasets makes it very hard for any gene to be consensually assigned to the same cluster.

### Phase Angles Analysis

Given that these 340 genes are considered cell-cycle regulated, the random-periods model (RPM) can be used to estimate their phase angles within the cell-cycle [33]. We provide an additional analysis of the results of the Bi-CoPaM method over the 340 genes by considering the distribution of their estimated phase-angles from the alpha-30 dataset. This additional analysis is detailed in the section “Phase Analysis of the Real Yeast Cell-Cycle Datasets” in File S1.

## Discussion

The Bi-CoPaM method allows one to obtain tunable clusters of different degrees of tightness. This is achieved by relaxing the conventional constraints at the clusters’ level and the genes’ level. Genes are allowed to be assigned to multiple clusters simultaneously, to be unassigned to any of the clusters, or to be assigned to one and only one cluster. Clusters are allowed to be tighter with fewer genes, or to be wider so they can overlap.

Table 4 shows an overview of the Bi-CoPaM method and many existing clustering methods, including the most commonly used ones. Each row of the Table represents one clustering method and each column represents one capability type. Each cell of the Table is filled with ‘YES’ or ‘NO’ to indicate if the corresponding clustering method has the corresponding capability or not.

**Table 4:** Comparison between Bi-CoPaM and many existing clustering methods.

Algorithms	Crisp clustering	Fuzzy clustering	Exclusive assignments	Un-assignments	Multiple-assignments	Consensus clustering	Tunable tightness	Multiple datasets
*k*-means	YES	NO	YES	NO	NO	NO	NO	NO
*k*-medoids (PAM)	YES	NO	YES	NO	NO	NO	NO	NO
Hierarchical	YES	NO	YES	NO	NO	NO	NO	NO
SOM	YES	NO	YES	NO	NO	NO	NO	NO
MCLUST	YES	NO	YES	NO	NO	NO	NO	NO
Fuzzy c means	YES	YES	YES	YES	YES	NO	NO	NO
Ensemble	YES	YES	YES	NO	NO	YES	NO	YES
SOON	YES	NO	NO	NO	NO	NO	NO	NO
Kernel *k*-means	YES	NO	YES	NO	NO	NO	NO	NO
Kernel FCM	YES	YES	YES	YES	YES	NO	NO	NO
SVC	YES	NO	YES	NO	NO	NO	NO	NO
Rough set	NO	YES	YES	YES	YES	NO	NO	NO
SSMCL	YES	NO	YES	NO	NO	NO	NO	NO
Bicluster	YES	NO	YES	NO	YES	NO	NO	NO
Bi-CoPaM	YES	YES	YES	YES	YES	YES	YES	YES

The description of the eight capabilities included in this Table is as follows: crisp clustering is the basic clustering in which each gene is assigned exclusively to one and only one cluster in a binary manner. Fuzzy clustering is assigning each gene to all of the clusters with fuzzy membership values whose total is unity. Exclusive assignments, un-assignments and multiple assignments are the three eventualities of a gene being exclusively assigned to one cluster, unassigned from all clusters, or assigned to multiple clusters simultaneously. Consensus clustering is the combination of various clustering results generated by various clustering methods into a single clustering result; some of the methods which fall in this class were mentioned in the introduction. Tunable tightness is the ability to provide clusters with different tightness levels; some examples are shown in **Table 3**. Multiple datasets capability is the capability of clustering the same set of genes from different microarray datasets and then combining them into a single consensus partition. Although we state in this Table the ensemble clustering in general has this capability, i.e. multiple datasets, this is because many ensemble clustering methods can do so theoretically. Yet to the authors’ knowledge, this has never been considered practically in the way in which the Bi-CoPaM does.

It can be seen in **Table 4** that only the Bi-CoPaM method possesses all of these capabilities. These capabilities of Bi-CoPaM arise from its diversity and flexibility. Diversity is achieved by its ability to provide clustering in consensus by using many different clustering methods and / or datasets. Flexibility is achieved by its ability to provide clustering in a variety of levels of tightness which is a key point in our proposed Bi-CoPaM. Binarization of the results of any simple fuzzy clustering method lacks diversity, while simple binarization of ensemble clustering results through the previously known techniques lacks flexibility. Most of the previous clustering methods, especially the ensemble ones, are considered as special cases in the new proposed paradigm. This means that the new paradigm generalises conventional clustering.

### Partition Generation

One way to generate partitions is to apply different methods with different parameters over the given dataset. Multiple runs can also be carried out if the methods are stochastic. This technique of partitions generation was used in most of the previous studies [39], [40] as well as in ours. In addition to that, we add another level of diversity by the usage of multiple microarray datasets to enhance diversity not only from the computational point of view (i.e. different clustering methods), but also from the biological point of view (i.e. different microarray datasets from different experiments). Amongst the other methods for partition generation found in the literature, subspaces of microarray datasets were generated by random sampling to enhance the diversity and the generation of the partitions [18], [41], [42].

### Relabelling

In many studies, the relabelling step has been investigated either as an independent step or fused with the consensus fuzzy partition generation step. In [42], the Hungarian method, whose complexity is 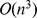, was used to solve the relabelling problem for ensemble clustering. This method was proposed originally by Kuhn to solve general assignment problems [43] and was applied in many different areas [30], [42]. A simpler method was used in [39] which is a greedy algorithm that constructs a pairwise similarity confusion matrix, finds the best matching column for each row, and then maps the two clusters that give the absolute best value to each other. The corresponding column and row for this value are removed from the matrix and the process is repeated until all clusters are mapped. This greedy behaviour can lead to a local optimum.

This method was called maximum greedy (MG) in [30], which proposed two methods - mean enhanced greedy (MEG) and single mean enhanced greedy (SMEG). These try to avoid local optima by giving poorly matched clusters the chance to be mapped first because the well matched clusters will still get good mapping later. Our min-max algorithm is like the first half of the MG algorithm used in [39] by finding the best matching column for each row, but then follows the approach of MEG (28) by matching the row and the column which make up the poorest match of these best matches.

### Number of Clusters

Many ensemble clustering methods aim at identifying the optimal number of clusters (*K*) inherently within the course of fusing different partitions with variable number of clusters such as in the weighted-association based method [21]. In [18], [42], their ensemble clustering methods do not support variable *K* values; they rather repeat the experiment with different *K* values then compare the results using a validation index to identify the optimal *K* value. Many other studies concentrate on developing other parts of ensemble clustering while using constant *K* values [39], [41].

In the Bi-CoPaM method, a constant number of clusters is used. This is to focus on the novelty proposed at the binarization step without distraction. To focus on the binarization part, we used synthetic datasets whose *K* values are predetermined and real yeast cell-cycle microarray datasets whose *K* values are suggested by the underlying biology [34]. Nonetheless, future work must be undertaken to design relevant validation techniques which can be used in validating the Bi-CoPaM’s results as well as in determining the optimum number of clusters.

### Binarization

Although the fuzzy consensus partition matrix (CoPaM) may serve as the output of ensemble clustering [39], in most cases the final output needs to be a binary CoPaM. The most widely used method for binarization is to assign each gene to the cluster in which it has the maximum membership value [25], [39], [41], which is equivalent to our proposed MVB. Other methods for binarization are like the efficient *O*(*n*) agglomerative algorithm based on the information bottleneck method which aims at finding the ‘most compressed summary’ binary partition matrix from the CoPaM, i.e. the binary partition matrix which preserves the maximum amount of information included in the CoPaM [14], [25].

In this paper, we have developed a complete framework for a novel paradigm at the binarization step in a modular way to be interfaced with any valid combination of the previous steps’ variants. We generalise the concept of clustering to allow any gene to be exclusively assigned to one cluster, simultaneously assigned to multiple clusters or unassigned from all of the clusters. Thus we not only propose advancing the way in which ensemble clustering is performed, but also propose enhancing the format of its ultimate results. It redefines the problem of clustering in general and the problem of binarization in specific. Moving to this new paradigm does not exclude the conventional one; it rather relaxes its constraints to make it more general while considering conventional clustering as a special case.

### Usefulness of Bi-CoPaM in Gene Discovery Research

The Bi-CoPaM paradigm does not treat all genes equally as happens in conventional binarization equivalent to MVB. Tuning the proposed binarization techniques to move far from MVB leads to more discerning treatments, i.e. the gene which was assigned to the same cluster by all of the clustering methods is treated significantly differently to the gene which was assigned to the same cluster by a majority, say 55%, of the methods. A researcher can tune this method to obtain tighter and purer clusters’ cores with low false-positive assignments so these genes can be considered for further biological experiments. A researcher can also tune binarization to obtain wider clusters which include all of the genes that might belong to them, i.e. low false-negatives. This might be needed in the studies in which the clusters’ cores are known and an extended view of the clusters with more potentially relevant genes is required.

The potentially interesting outcomes of Bi-CoPaM go beyond the genes contained in the resulting clusters themselves. Focusing on the subset of genes which is not assigned to any of the clusters or which is multiply-assigned to more than one cluster simultaneously can be of research importance and have biological meaning.

### Multiple-Datasets vs. Single-Dataset

From the biological literature, it can be found that the yeast gene YAL040C / CLN3 is a cyclin, i.e. a gene which shows cyclical accumulation during particular phases of the cell-cycle, which is involved in the G1 phase cell-cycle progression operations such as the regulation of many other G1 cyclins like CLN1 and CLN2 [44].

**Figure 6:**
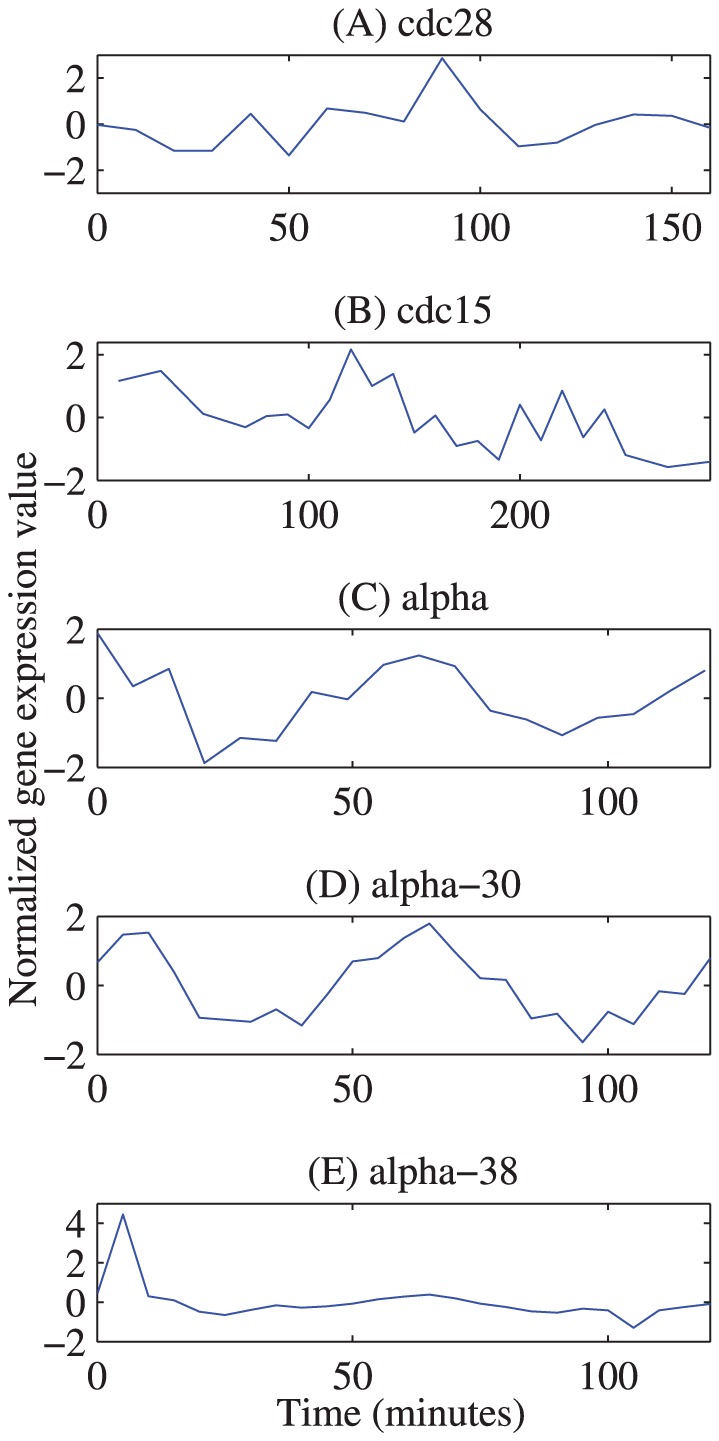
YAL040C / CLN3 gene expression profiles in the five microarray datasets. Genetic expression profiles for the cyclin CLN3 from the five datasets cdc28, cdc15, alpha, alpha-30 and alpha-38 are plotted. Although the gene’ is known to be expressed periodically, different levels of periodicity for its profiles can be seen for different datasets clearly.

**Figure 6** shows the normalised gene expression profiles of this gene from the five yeast datasets considered in this study. The profiles in the datasets cdc15, alpha and alpha-30 are just as expected, in that each of them shows two high-expression regions at the G1 stage from the two cell-cycles. The expression profile in cdc28 is fine in that it shows a very obvious peak at the G1 stage from the second cycle. The profile in alpha-38 is far from what is expected because the expression at the second time point (at 5 minutes) has, for some reason, a large positive impulse which flattens the normalised expression profile at other time points.

If the genes were clustered only according to their profiles in the dataset alpha-38, the gene CLN3 will not be clustered with the G1 phase genes. On the other hand, the fuzzy membership values of this gene in the five clusters (C1) to (C5), which are shown in Table 5, clearly show the power of applying the Bi-CoPaM method over multiple, relatively noisy, datasets. The gene is assigned to the cluster (C1), which represents the early G1 phase, with a membership value of 0.63, while the closest competitor is (C5) with the membership of 0.17. Thus, despite having poor expression profiles in some datasets, this gene is included in the correct biological cluster (C1) in most of the binarization configurations. Here CLN3 has been used as an example; though, many other examples are found within these 340 genes that have relatively poorer profiles in one or more of the five datasets.

**Table 5:** Fuzzy membership values for the CLN3 gene.

Cluster	C1	C2	C3	C4	C5
Fuzzy membership	0.63	0.02	0.02	0.16	0.17

### Summary and Future Work

We propose a new gene clustering paradigm which allows each gene to be assigned exclusively to one cluster, assigned simultaneously to multiple clusters, or assigned to no clusters. Although this four-step paradigm differs significantly from the conventional ones, in this study we adopted some published techniques for the first three steps to help us elucidate and evaluate the novel and important fourth step of binarization techniques.

The modularity of the proposed binarization step makes it a straightforward task to develop research in many directions. For example, more sophisticated variants of the techniques used in the first three steps to tackle different issues such as the diversity of the generated individual partitions, the optimal number of generated clusters, and the optimal relabelling solution can be simply incorporated. More importantly, novel techniques can easily replace the binarization module and preserve the paradigm.

Future work will address the problem of validation by designing novel validation indices or techniques so that Bi-CoPaM can be compared with its future variants. Moreover, Bi-CoPaM can comprehensively analyse the same set of genes from different microarray datasets. Such analysis has been carried out in some studies [36], [45] without a clustering approach and its importance has been demonstrated in this study through the analysis of a set of yeast genes from five different microarray datasets. This suggests that future gene discovery studies can benefit from using Bi-CoPaM to cluster the profiles of the same set of genes from different datasets.

## Supporting Information

File S1 **Additional analysis for synthetic as well as real yeast cyclic datasets by the random periods model (RPM).** This Supplementary File consists of two main sections. The first section provides the details of the experimental design as well as the results of a fairly comprehensive additional Bi-CoPaM experiment over a different synthetic dataset generated based on the RPM model. Separately, the second section shows an application of the RPM model to the results of our analysis of real yeast cell-cycle datasets in the main text; this provides additional validation to them, and therefore demonstrates the usefulness of the Bi-CoPaM method in such cases.
(PDF)
