# Supplementary material for: Paradigm of Tunable Clustering Using Binarization of Consensus Partition Matrices (Bi-CoPaM) for Gene Discovery
Source: PLoS One. 2013 Feb 11;8(2):e56432. doi: 10.1371/journal.pone.0056432 (PMC3569426; doi:10.1371/journal.pone.0056432)
Supplement: File S1 — Additional analysis for synthetic as well as real yeast cyclic datasets by the random periods model (RPM). This Supplementary File consists of two main sections. The first section provides the details of the experimental design as well as the results of a fairly comprehensive additional Bi-CoPaM experiment over a different synthetic dataset generated based on the RPM model. Separately, the second section shows an application of the RPM model to the results of our analysis of real yeast cell-cycle datasets in the main text; this provides additional validation to them, and therefore demonstrates the usefulness of the Bi-CoPaM method in such cases. (PDF) [file pone.0056432.s001.pdf]

Supplementary File S1

Random Periods Model (RPM) Synthetic Data Analysis

We support our discussion about the Bi-CoPaM method by exploring its application over a second, more realistic, synthetic dataset. This dataset is based on the more realistic model of cell-cycle regulated genes, the cyclic random periods' (RPM) model, proposed by Liu and colleagues [1]. This section explains the RPM model, the generation of the synthetic dataset, the experimental procedure of Bi-CoPaM clustering, and the results.

The RPM Model

Liu and colleagues proposed the *random periods model (RPM)* for the expression profiles of cell-cycle regulated genes [1]. This model accounts for the attenuation noticed in these cyclic expression profiles due to the asynchrony that occurs between the cells in the considered population [1]. For the gth gene, the expression value is Y_g and is expressed by the model:

$$Y_g = f(t, \theta_g) + \varepsilon_g(t) \qquad [1]$$

$$f(t, \theta_g) = a_g + b_g t + \frac{K_g}{\sqrt{2\pi}} \int_{-\infty}^{\infty} \cos\left(\frac{2\pi t}{T e^{\sigma z}} + \phi_g\right) e^{-\frac{z^2}{2}} dz \qquad [2]$$

$$\theta_g = (K_g, T, \sigma, \phi_g, a_g, b_g). \qquad [3]$$

This expression value consists of the modelled expression $f(t, \theta_g)$ and an additive zero mean random number about which no further assumptions are imposed. The function f depends on the time of expression (t) and a set of gene-dependent parameters θ_g.

The function in equation [2] has two main parts – the linear skewing part and the main cyclic part. The second part follows the basic idea of this model that the cyclic expression of a specific gene at some point in time is the integration (or summation) of slightly asynchronous cosinusoidal expressions. In this integration, it can be seen that T is the period of the cycle, ϕ_g is the phase, and the value of σ controls the rate of

attenuation of the resultant expression profile. The first part, which is represented by $(a_g + b_g t)$, adds a linear skewness to the cosinusoid. Here a_g is the y-intercept and b_g is the slope. The two parameters T and σ are considered common to all of the genes within the same dataset, so they are not given the gene-specific subscript g.

An R language code was developed by the authors who proposed the model to estimate this set of parameters given the expression profiles of a set of genes is available at http://www.niehs.nih.gov/research/resources/software/biostatistics/r-code/index.cfm.

Data and Parameters Estimation

Recently, Fernandez and colleagues listed 34 *Saccharomyces cerevisiae* cell-cycle regulated genes which serve as signature genes for the cell-cycle phases [2]. The distribution of these genes over their labelled cell-cycle phases is: Eleven genes in G1, seven in S, eight in G2, five in M, and one in each of the three transitions G1/S, S/G2 and M/G1. We consider the 31 genes mapped to G1, S, G2 and M in this study.

The parameters of the RPM model for these 31 genes were estimated based on their profiles in the alpha-30 dataset provided by Pramila and colleagues [3], and they are listed in Table S1. It can be seen in this Table that the phase values for each of the four cell-cycle phases are clearly close to each other. The only exception is the DSE4 gene.

Table S1. Estimated RPM parameters for the 31 cell-cycle regulated genes from the alpha-30 dataset.

Cell-cycle phase	Gene symbol	$\hat{K}$	$\hat{T}$, minutes	$\hat{\sigma}$	$\hat{\Phi}$, radian	$\hat{a}$	$\hat{b}$	Cell-cycle phase	Gene symbol	$\hat{K}$	$\hat{T}$, minutes	$\hat{\sigma}$	$\hat{\Phi}$, radian	$\hat{a}$	$\hat{b}$
G1 (11 genes)	CLN2	0.45	60.00	0.100	3.67	0.000	0.000		HTZ1	0.18	81.85	0.162	3.47	-0.104	0.001
	RFA1	0.38	61.69	0.088	4.07	-0.097	0.001		KIP3	0.13	60.00	0.100	2.36	0.000	0.000
	MSH6	0.45	60.00	0.148	3.89	-0.011	0.000	G2 (8 genes)	CDC5	0.43	74.23	0.143	1.75	-0.094	0.002
	MCD1	0.80	61.33	0.157	3.95	-0.169	0.001		ASE1	0.36	74.00	0.137	1.51	0.055	-0.001
	RAD51	0.47	70.00	0.200	4.19	0.000	0.000		CHS2	0.47	72.59	0.070	1.48	-0.300	0.005
	SMC3	0.41	60.52	0.142	3.87	-0.059	0.000		HOF1	0.43	70.00	0.100	1.05	0.000	0.000
	POL2	0.33	60.01	0.132	3.74	-0.055	0.001		SWI5	0.37	76.32	0.131	2.01	-0.069	0.001
	RNR1	0.64	60.00	0.100	3.93	0.000	0.000		MOB1	0.26	70.00	0.100	1.57	0.000	0.000
	POL1	0.40	60.00	0.100	3.67	0.000	0.000		BUD4	0.51	74.36	0.176	1.87	-0.162	0.003
	MRC1	0.24	60.00	0.100	3.93	0.000	0.000		MYO1	0.28	70.00	0.100	1.57	0.000	0.000
	DSE4	0.46	76.95	0.000	0.29	-0.876	0.007	M (5 genes)	DBF2	0.24	60.00	0.010	5.76	0.000	0.000
S (7 genes)	HTA2	0.71	59.31	0.122	2.56	-0.369	0.003		CDC20	0.26	68.19	0.077	0.99	-0.100	0.001
	HHT1	0.50	57.82	0.109	2.69	-0.189	0.002		KIN3	0.28	72.93	0.000	0.98	-0.102	0.002
	HTB2	0.68	59.71	0.125	2.75	-0.305	0.002		SST2	0.89	69.74	0.730	0.38	-0.060	-0.002
	HHT2	0.60	60.00	0.100	2.62	0.000	0.000		CDC6	0.26	60.00	0.100	5.50	0.000	0.000
	HHF1	0.69	57.93	0.123	2.60	-0.280	0.002								

Synthetic Data Generation

The distribution of each of the parameters was inferred from these real-data-based estimations. Some statistical features of these estimations are shown in Table S2. These summarized statistical estimations were used to generate a synthetic dataset which shows the expression profiles of 620 synthetic genes over 25 time-points covering two hours with five-minute gaps between each two points. The genes were synthesized such that they belong to four clusters representing the four cell-cycle phases G1, S, G2 and M. The number of genes considered for each of these clusters was proportional to the number of genes which represent the corresponding phase in Table S1, namely, 220, 140, 160 and 100 genes in the clusters representing G1, S, G2 and M, respectively.

Clustering Experiment Procedure

The 620 synthesized genes' expression profiles were clustered into four clusters by k-means with Kauffman deterministic initialization [4], self-organizing maps (SOMs) with bubble and Gaussian neighborhoods, hierarchical clustering (HC) with complete and Ward linkage methods, and self-organizing oscillator networks (SOON) with all of the $[b, C_E, d_0]$ parameters combinations produced by $b \in \{0.1, 1, 50, 100\}$, $C_E \in [0.1: 0.02: 0.2]$, and $d_0 \in [2.5: 0.1: 3.5]$. Fifty five partitions out of the 264 generated SOON partitions actually produced four clusters, and they were relabeled and combined into one CoPaM which was then combined with the other partitions from the rest of the methods to produce the final CoPaM.

The six binarization techniques IB, UB, VTB, DTB, TB and MVB were applied over the final CoPaM with different values of parameters to generate a set of final binary partitions with different levels of tightness / wideness.

Results

The numbers of genes included in each of the four clusters at each adopted binarization configuration (technique and parameter) are listed in Table S3. The results in the Table are organized into two tracks of binarization configurations, each of which starts from very wide clusters which are tightened gradually until they reach the tightest case.

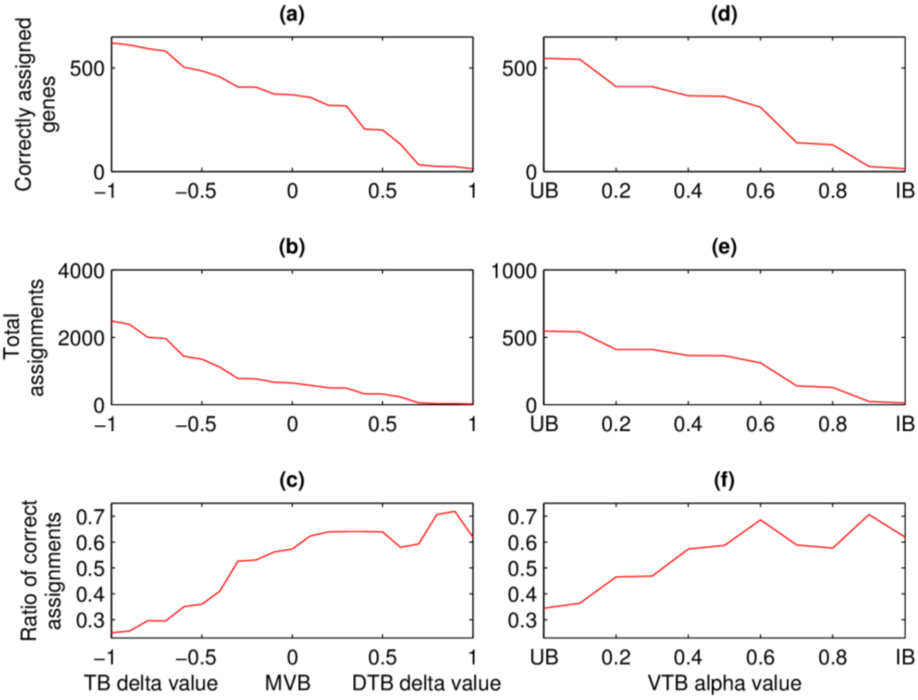

Figure S1. Correctly assigned genes in the RPM synthetic data Bi-CoPaM results.

Subplots (a) and (d) show the number of correctly assigned genes, (b) and (e) show the total number of assigned genes, and (c) and (f) show the ratios of correctly assigned genes to total assignments. (a), (b) and (c) are for the TB-MVB-DTB track cases, and (d), (e) and (f) are for the UB-VTB-IB track cases.

Correct Assignments Analysis

Figure S1 shows the numbers of correct assignments, total assignments, and the ratio of correct assignments to total assignments for the binarization cases from both tracks, namely TB-MVB-DTB and UB-VTB-IB. Note that, in the best cases, correct assignments barely exceed 70% of total assignments. This is significantly lower than what was found in the results of the 60 cyclic synthetic datasets generated by the non-attenuating model, where many cases reached 100%.

This difference is mainly because of many real-datasets issues that are captured by the RPM synthetic dataset but not captured by the other model. One major issue is that the phases of the clusters in the non-attenuating model are equally distributed over the 2π period while they are allocated at their real-data-based estimations in the RPM model. Figure S2 shows the probability distribution functions for the phase values of the four clusters as estimated by the RPM model and shown in Table S2.

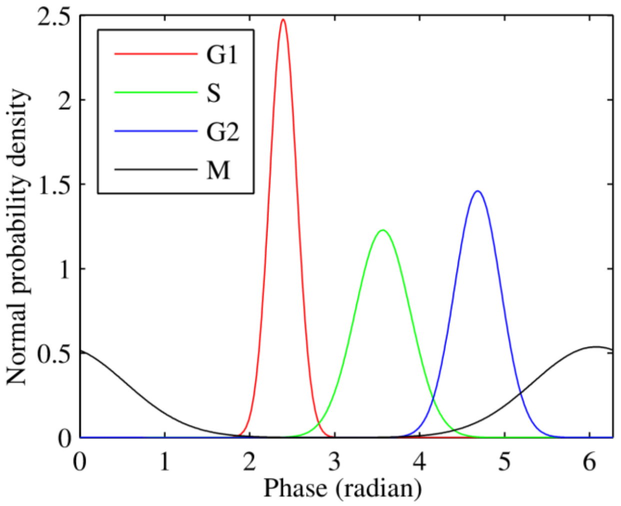

Figure S2. Normal probability density functions for the four clusters based with mean and standard deviation values as estimated by the RPM model.

This Figure shows that the distribution of the four clusters' phases is not uniform over the 2π period. It can be seen that the G1 phase cluster has the least phase variations and the least amount of overlapping with the other clusters. On the other hand, the G2 phase cluster has significant overlaps with both M phase and S phase clusters. This does not only explains why correct assignments did not reach very high percentages as in the non-attenuation model's case, it also conforms to the relative levels of resistance to losing genes while tightening for the four clusters as in the results in Table S3. In other words, G1 phase cluster preserves more genes when tightened when compared to any other cluster, while G2 preservation is the least.

Table S2. Summary statistical features of the real-data-based yeast cell-cycle RPM estimations.

Statistical Feature	$\hat{K}$	$\log(\hat{K})$	$\hat{T}$, minutes	$\hat{\sigma}$	Trimmed $\hat{\sigma}^*$	$\hat{a}$	$\hat{b}$	$\varepsilon^{\neq}$
Mean	0.44	-0.92	65.8	0.13	0.12	-0.11	0.0011	0
Standard deviation	0.18	0.44	7.1	0.12	0.03	0.18	0.0017	0.14
Distribution	Lognormal	Normal	Normal	Normal but has outliers	Normal	Normal	Normal	Laplacian

	G1&	S	G2	M
$\hat{\phi}$ mean	2.39	3.57	4.68	6.08
$\hat{\phi}$ standard deviation	0.16	0.32	0.27	0.74

* The values of $\hat{\sigma} > 0.7$ and $\hat{\sigma} < 0.05$ were trimmed out because they are clearly outliers. The outliers are three genes, DSE4, KIN3 and SST4, and as can be seen when trimmed and untrimmed cases are compared in this table, these outliers affect the value of the standard deviation significantly if not trimmed.

≠ The distribution of the additive error (ε) was taken from a much larger set of genes than these 31 genes. The most periodic thousand yeast genes listed by Pramila and colleagues were used to derive a more general distribution of this error term [3]. The actual measured mean for this parameter was (-0.009) and the assumed mean by the model is zero. Thus, we assume it zero here.

& The phase mean and standard deviation of G1 genes are calculated while excluding the phase value of the outlier gene DSE4.

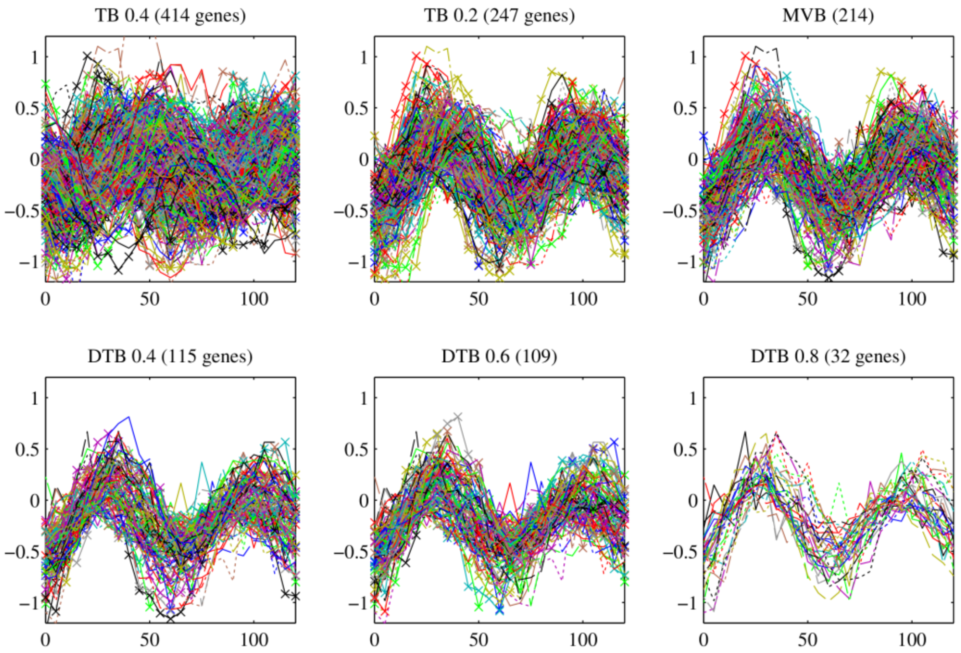

Figure S3. S phase cluster (C2) members profiles at different gradually increasing levels of tightness.

The x-axis for each of the six sub-plots represents time in minutes, and the y-axis represents the genetic expression. All y-axes are set to the same limits so that the plots can be directly comparable.

Tunable Tightness Effect on Clusters

Figure S3 shows the profiles of the genes included in the S phase cluster (C2) at the wide and overlapping clusters' case of TB 0.4 and then at gradually increasing levels of tightness towards the tight case of DTB 0.8. It is clear from this figure that tightening the cluster generally results in less numbers of genes that are closer to the core of that cluster. The MVB result looks noisy for this specific cluster, while the result at DTB 0.6 and maybe a 0.4 are reasonable. Cases in which only few genes are included in a cluster, such as the case at DTB 0.8 here, are not usually meaningful.

Table S3. Assignment of genes from the RPM synthetic dataset by Bi-CoPaM

	Bin. tech.	Param. (δ)	Genes in clusters				Bin. tech.	Param. (α)	Genes in clusters			
			C1 (G1)	C2 (S)	C3 (G2)	C4 (M)			C1 (G1)	C2 (S)	C3 (G2)	C4 (M)
Wide	TB	0.8	617	511	449	425	UB	-	620	351	333	282
	TB	0.6	501	368	296	272	VTB	0.1	613	306	329	237
	TB	0.4	414	267	255	177	VTB	0.2	293	232	203	153
	TB	0.2	247	161	205	154	VTB	0.3	289	232	202	152
	TB	0.1	230	153	159	124	VTB	0.4	231	141	154	111
Complementary	MVB	-	214	152	159	121	VTB	0.5	212	141	154	111
	DTB	0.1	199	137	127	111	VTB	0.6	195	117	94	46
	DTB	0.2	195	136	98	70	VTB	0.7	111	86	13	26
	DTB	0.4	115	108	51	46	VTB	0.8	106	83	7	26
	DTB	0.6	109	83	8	26	VTB	0.9	32	1	1	0
Tight	DTB	0.8	32	1	1	0	IB	-	21	0	0	0
	**Track 1 (TB-MVB-DTB)**						**Track 2 (UB-VTB-IB)**					

Phase Analysis of the Real Yeast Cell-Cycle Datasets

This section provides an additional analysis of the results of applying the Bi-CoPaM method over 340 genes from five real yeast cell-cycle datasets. This analysis is based on investigating the agreement between the estimated phase angles of the genes and their assignments to the clusters by the Bi-CoPaM method.

The RPM model explained in the previous section was used to estimate the phase angles ($\hat{\phi}$) for the 340 yeast genes which were considered in our main paper's analysis. This estimation was based on their profiles in the alpha-30 dataset. The distribution of these phase angles within each of the five clusters C1 to C5 at the binarization configurations MVB, DTB 0.2, DTB 0.4 and DTB 0.6 are shown in Figure S4.

The most obvious observation in this figure is that the clusters C1 to C4 occupy distinct regions of phases, and when clusters are tightened the distinctness of these regions increase. The other major observation is that the C5 cluster does not occupy any specific region of phases; this cluster has already been shown as the least distinct in the Bi-CoPaM results as it lost its members quickly while tightening.

Figure S5 shows the average profiles from the alpha-30 dataset for the five clusters at MVB binarization. As can be seen in this Figure, clusters C1 to C4 show clear cyclic behaviour with two cycles covering the 120 minutes period; each of them has its distinct phase-shift. On the other hand, C5 cluster's distinctness is in its relatively high peak at the second time point and not in its specific cell-cycle phase-shift. The genes of this cluster also show some shallower peaks at other time points. These shallow peaks, which overlap with some peaks of the C1 to C4, made the genes within the C5 cluster less specific to this cluster and thus more compatible by other clusters. Moreover, this acyclic behaviour C5 cluster made it totally undetectable by merely looking at its genes estimated phases, e.g. as in Figure S4, yet it is detectable by sophisticated clustering algorithms. Thus, although we have shown the agreement between the Bi-CoPaM results and the phase estimations for four cyclic clusters, which supports the validity of the Bi-CoPaM method, we have also shown that mere phase-based analysis might not be generalized as an alternative to clustering, especially for acyclic genes.

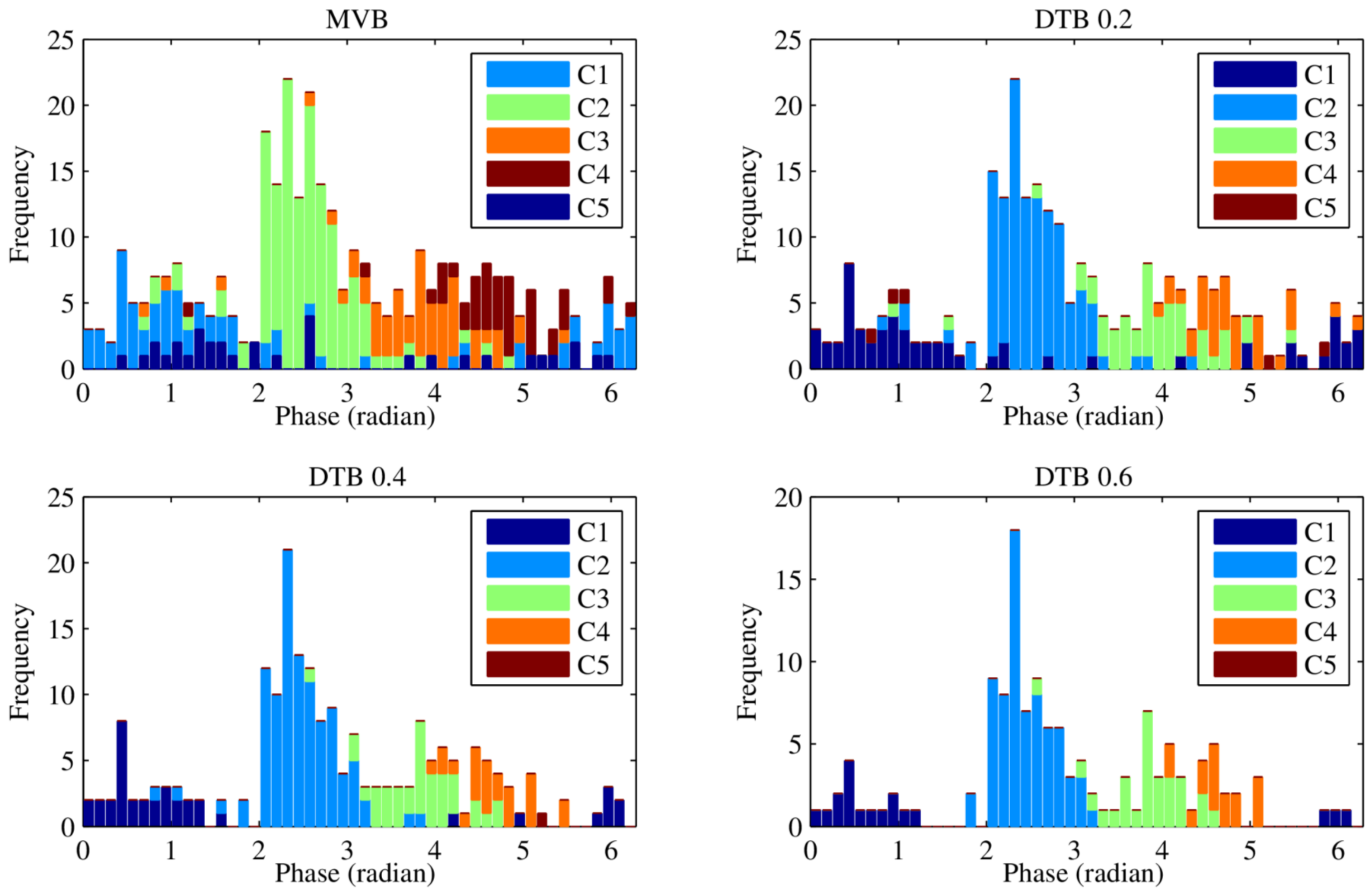

Figure S4. Bar plots showing the distribution of phase angles of the genes included in the five Bi-CoPaM clusters at four different tightness levels.

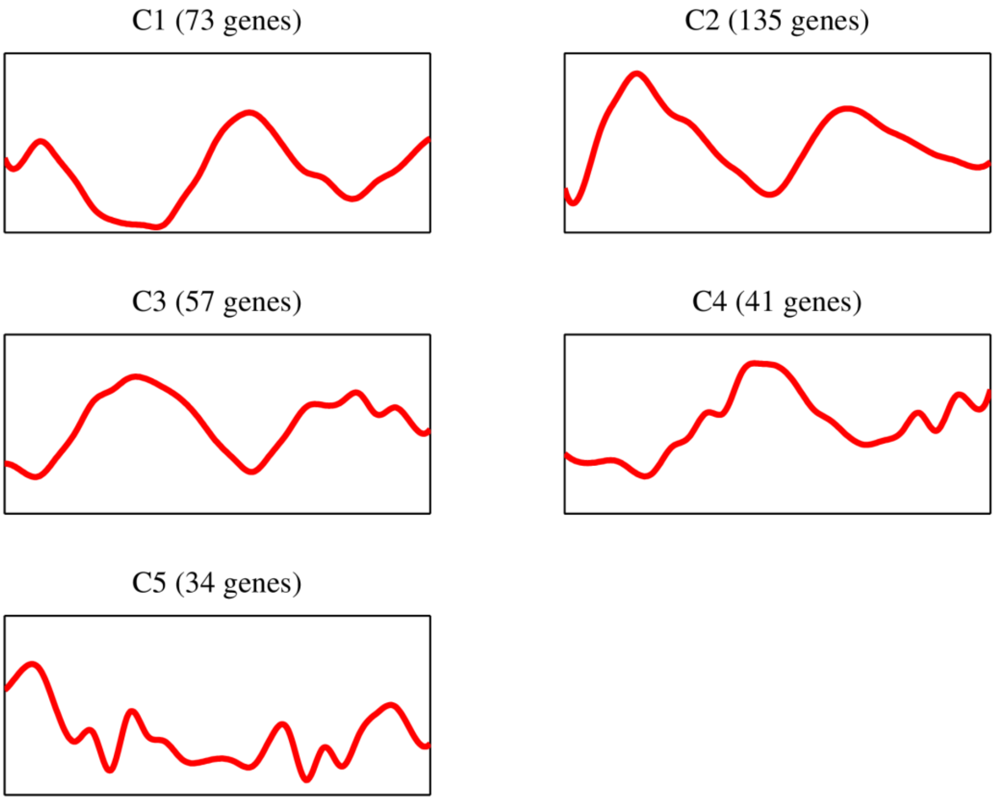

Figure S5. Average profiles for the 340 genes five clusters from the alpha-30 dataset at MVB.

The axes labels have been concealed for clarity; for each sub-plot, the x-axis represents time (0 to 120 minutes), and the y-axis represents the average gene expression. The average profiles were smoothed by spline interpolation for better presentation.

References

1. Liu D, Umbach DM, Peddada SD, Li L, Crockett PW, Weinberg CR (2004) A random-periods model for expression of cell-cycle genes. PNAS 11:7240-7245.

2. Fernandez MA, Rueda C, Peddada SD (2012) Identification of a core set of signature cell cycle genes whose relative order of time to peak expression is conserved across species. Nucleic Acids Research 40:2823–2832.

3. Pramila T, Wu W, Miles S, Noble WS, Breeden LL (2006) The Forkhead transcription factor Hcm1 regulates chromosome segregation genes and fills the S-phasegap in the transcriptional circuitryof the cell cycle. Genes and Development 20:2266–2278.

4. Pena JM, Lozano JA, Larranaga P (1999) An empirical comparison of four initialization methods for the K-Means algorithm. Pattern Recognition Letters 20:1027-1040.
